# Digital therapy using dichoptic visual perceptual learning to improve stereopsis in children with intermittent exotropia

**DOI:** 10.3389/fdgth.2026.1738858

**Published:** 2026-03-09

**Authors:** Eun Namgung, Hyeongsuk Ryu, Jin Woong Lee, Byung Joo Lee, Dong-Wha Kang

**Affiliations:** 1 Asan Institute for Life Scienceş Asan Medical Center, Seoul, Republic of Korea; 2 Department of Ophthalmology, Asan Medical Center, University of Ulsan College of Medicine, Seoul, Republic of Korea; 3 Department of Neurology, Asan Medical Center, University of Ulsan College of Medicine, Seoul, Republic of Korea; 4 Nunaps Inc., Seoul, Republic of Korea

**Keywords:** dichoptic stimulation, digital therapy, intermittent exotropia, virtual reality, visual perception learning

## Abstract

**Background:**

Intermittent exotropia impairs binocular vision and stereopsis in children, and visual perceptual learning (VPL) with dichoptic stimulation offers a potential therapy. This prospective exploratory study aimed to evaluate the efficacy of an 8-week at-home dichoptic VPL program delivered through virtual reality (VR)-based digital therapy in improving stereopsis and binocular sensory function in children with intermittent exotropia.

**Method:**

Children aged 6–16 years diagnosed with intermittent exotropia (*n* = 18) completed training at least 10 min per day, five days per week, using VR content tailored to their deviation angle. Outcomes included near stereoacuity (Titmus Stereo Test) and distance binocular sensory status (Worth 4 Dot test), measured at baseline and 8 weeks.

**Result:**

Stereoacuity improved significantly across all participants [mean change: −0.21 log arcsec; 95% confidence interval (CI), −0.28 to −0.13; *P* < 0.001], with greater improvement in a subgroup with subnormal baseline stereoacuity (mean change: −0.31 log arcsec; 95% CI, −0.39 to −0.23; *P* < 0.001). The odds of achieving sensory fusion increased significantly [odds ratio (OR) = 5.50; 95% CI, 1.38–22.0; *P* = 0.016], and 44.4% of participants showed transition to fusion. In the subnormal subgroup, 62.5% achieved fusion (OR = 21.0; 95% CI, 1.72–258.0; *P* = 0.017).

**Conclusion:**

These findings suggest VR-based dichoptic VPL as a promising and personalized digital therapy to enhance binocular vision in pediatric intermittent exotropia, particularly in cases with subnormal stereoacuity.

## Introduction

Stereoscopic vision, defined as depth perception based on retinal disparity, represents the most advanced form of binocular function (1). Reduced stereopsis is commonly associated with conditions such as strabismus, an ocular misalignment that disrupts binocular coordination and may lead to suppression of input from one eye (2). Intermittent exotropia (IXT), the most common form of childhood strabismus, is characterized by episodic outward deviation of one eye and significantly affects quality of life by impairing binocular vision, particularly at a distance (3, 4).

The primary goal of IXT treatment is to restore ocular alignment and preserve binocular vision, including stereopsis. Conventional non-surgical approaches primarily involve monocular use and do not actively promote binocular fusion (5–7). While surgical correction typically achieves anatomically normal alignment in the early postoperative period, individuals with reduced stereoacuity show higher recurrence rates due to impaired binocular integration (8, 9). Targeting functional plasticity of the brain has been proposed as a promising strategy to enhance stereopsis and improve both strabismus outcomes and surgical prognosis (10, 11).

Recent studies have identified visual perceptual learning (VPL) as an effective method for long-term vision improvement by enhancing visual cortex plasticity through repeated training (12–14). Virtual reality (VR)-based digital therapy using head-mounted displays is emerging as a promising approach for binocular vision training in children, providing an immersive environment with precise control of head movements while minimizing side effects and spatial or temporal constraints (15, 16). Dichoptic VPL, which presents different images to each eye simultaneously, strengthens stereoscopic vision by stimulating the visual cortex and promoting image fusion (17, 18).

We developed a personalized VR-based digital therapy for children with IXT, incorporating an 8-week dichoptic VPL program designed to enhance stereopsis and binocular sensory function. The program included deviation angle assessment and customized dichoptic training tailored to each child's ocular deviation, aiming to improve alignment and depth perception through VPL. Efficacy was assessed by comparing near stereoacuity and distance binocular sensory status at baseline and after 8 weeks using standardized ophthalmologic evaluations.

## Method

### Participants

Between October 26, 2021 and October 11, 2022, 20 patients diagnosed with IXT were recruited from the Ophthalmology Department of Asan Medical Center. Inclusion criteria were as follows: age between 6 and 16 years; a diagnosis of IXT confirmed by a board-certified ophthalmologist (B.J.L.); and the ability to use a virtual reality head-mounted display (VR-HMD) without difficulty.

Exclusion criteria included the presence of organic ocular disease or neurological abnormalities; amblyopia or anisometropia exceeding 2.0 diopters (D) in spherical equivalent; abnormal retinal correspondence; vertical strabismus exceeding 4 prism diopters (PD); a history of ophthalmic surgery; recent (≤1 month) or ongoing occlusion therapy; and a history of prematurity (gestational age <37 weeks or birth weight <2,500 × g).

The study protocol was approved by the Institutional Review Board of Asan Medical Center (IRB No. 2021-0650) and conducted in accordance with the principles of the Declaration of Helsinki. Written informed consent was obtained from the guardians of all minor participants after they were fully informed of the study procedures and potential risks.

### Study design

Clinical assessments included demographic characteristics, medical and medication history, vital signs, and physical examination. Ophthalmologic evaluations comprised measurements of spherical equivalent refractive errors and best-corrected visual acuity (BCVA). IXT was classified as basic type (distance–near deviation difference <10 PD) or convergence-insufficiency (CI) type (difference ≥10 PD). The primary outcome measures were near stereoacuity and distance binocular fusion.

**Figure 1:**
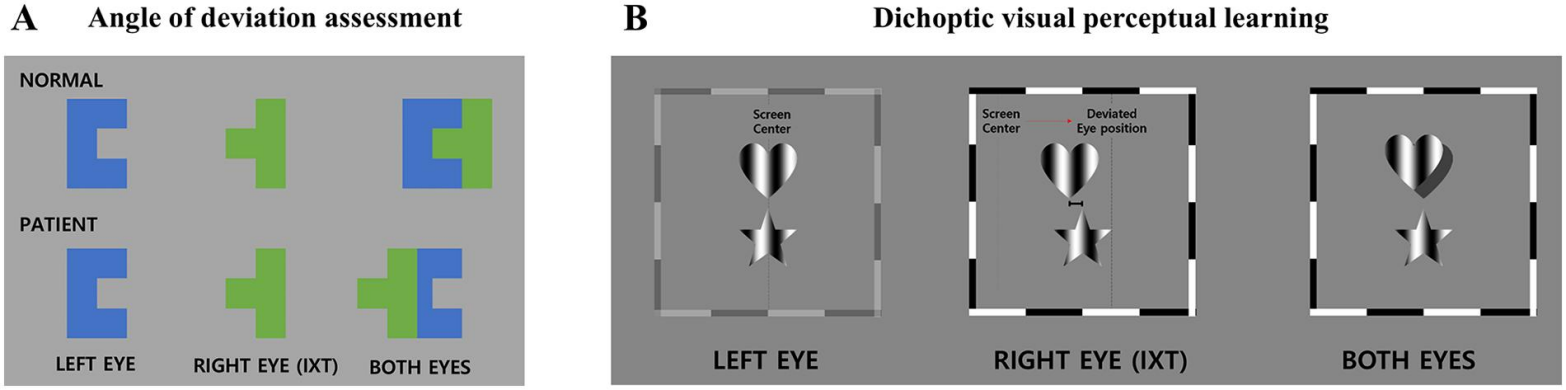
Virtual reality-based digital therapy delivering 8-week dichoptic visual perceptual learning. Overview of the dichoptic visual perceptual learning program provided by a virtual reality-based digital therapy over an 8-week period. **(A)** Angle of deviation assessment task used to quantify ocular misalignment through dichoptic image presentation. **(B)** Personalized dichoptic visual perceptual learning task, which presents separate images to each eye based on the individual's angle of deviation to progressively train and improve stereopsis. IXT, intermittent exotropia.

Participants underwent dichoptic VPL training at home using a standalone VR-HMD with two main components (Figure 1). First, the angle of ocular deviation was quantified using a digital binocular stimulation system with dichoptic presentation (Figure 1A). Next, participants completed approximately 70 trials per 10-minute session, five times per week, over an 8-week period. Stimuli were individually adjusted based on each participant's deviation angle to elicit depth perception (Figure 1B).

Following the training period, ophthalmologic assessments were repeated to evaluate changes in near stereoacuity and distance binocular sensory status.

### Ophthalmic examination

Baseline ophthalmologic assessments included cycloplegic refraction and BCVA. Cycloplegia was induced using either 1% cyclopentolate (Cyclogyl) or 0.5% atropine to temporarily inhibit accommodation and enable accurate measurement of refractive error. Spherical equivalent and BCVA values were recorded, with BCVA expressed in logarithm of the minimum angle of resolution (LogMAR) units.

Outcome measures included near stereoacuity and distance binocular sensory status, evaluated at baseline and after 8 weeks of dichoptic VPL training. Near stereoacuity, expressed in logarithm of arcseconds (log arcsec), was measured using the Titmus Stereo Test at a 40 cm viewing distance while participants wore polarized glasses and full refractive correction. Stereoacuity values ranged from 20 to 4800 arcseconds, with lower values indicating finer depth discrimination and better stereopsis.

Distance binocular sensory status was assessed using the Worth 4-Dot (W4D) test at a 6 m distance. Participants wore red–green anaglyphic glasses, and the test was administered twice to ensure response consistency. Results were categorized as follows: fusion (perception of four dots, indicating normal binocular function), diplopia (five dots, indicating misalignment), or suppression (two or three dots, indicating cortical suppression of one eye, typically as a compensatory mechanism or sign of amblyopia).

### Dichoptic visual perceptual learning

Participants performed dichoptic VPL training at home using a standalone VR-HMD (Oculus Quest 2; Facebook, Menlo Park, CA) for at least 10 min per session (approximately 70 trials), five times per week over 8 weeks. The device featured a 120-Hz refresh rate, 1832 × 1,920 per-eye resolution, and adjustable interpupillary distance (IPD; 58, 63, or 68 mm) to ensure optimal optical alignment.

The VR-based dichoptic VPL program was developed using the Unity game engine (Unity 2022.3.13f1) with the Oculus Integration SDK (v57.0). Visual stimuli were rendered against a uniform mid-gray background (RGB: 127, 127, 127), while stimulus elements were presented in a fixed blue-green color (RGB: 0, 119, 140). All color parameters were held constant across sessions and participants.

The program consisted of two components: (1) measurement of ocular deviation to quantify binocular misalignment and (2) personalized dichoptic training adjusted to each participant's deviation angle to enhance stereopsis (Figure 1).

At the beginning of each daily session, participants completed a calibration procedure to align dichoptic images. Distinct images were presented separately to each eye at the central fixation point through the VR-HMD. The images (“ㄷ” and “ㅓ”) aligned to form a square only when the eyes were properly aligned. Participants with ocular misalignment used the VR-HMD controller to adjust the position of each image until the “ㄷ” and “ㅓ” characters merged into a square configuration, indicating perceptual alignment. This calibration procedure was repeated three times per session. The pixel displacement required for alignment was converted into PD (Δ), representing the angle of deviation (Figure 1A).

IPD was set using the hardware adjustment of the Oculus Quest 2 and was not modified programmatically. Head position and eye height were defined using the device's default central reference frame, without additional software-based height correction. Visual stimuli were presented in a virtual environment at a fixed viewing distance of 2 m, with a constant angular size of 12 degrees.

For the training task, identical shapes were presented simultaneously to the dominant and strabismic eyes at corresponding spatial positions. A horizontal positional disparity was introduced in the image presented to the strabismic eye, calibrated to the individual deviation angle measured during calibration. This manipulation generated binocular disparity cues to elicit depth perception. Using the VR-HMD controller, participants indicated which of the two shapes (top or bottom) appeared more convex, i.e., protruding in depth (Figure 1B).

Task difficulty was modulated using a 2-down 2-up staircase procedure in real time based on performance. As performance improved, the binocular disparity between shapes was progressively reduced to present finer disparity cues, maintaining task difficulty near perceptual threshold. Stereoacuity thresholds were continuously estimated in arcseconds based on task performance, and training parameters were dynamically updated to support individualized learning trajectories.

### Outcome measures

The primary outcome measure was change in near stereoacuity, measured using the Titmus Stereo Test. Stereoacuity was analyzed using changes in log arcsec values and corresponding octave steps. (Step 0: <0.3 log arcsec; Step 1: 0.3–<0.6 log arcsec; Step 2: ≥0.6 log arcsec; Table 1). This standardized logarithmic, step-based approach enables accurate representation of clinically meaningful changes, with a ≥2-octave-step (≥0.6 log arcsec) improvement predefined as the criterion for significance (19, 20).

**Table 1:** Classification of stereoacuity change using octave steps and log arcsecond values.

Change in octave steps	Corresponding Change in log arcsec	Interpretation	Total (*n* = 18)	Subnormal (*n* = 8)
≥ + 2 octave steps	≥ + 0.6	Clinically meaningful improvement	0 (0%)	0 (0%)
≥ + 1 and < + 2 octave steps	≥ + 0.3 and < + 0.6	Mild to moderate improvement	6 (33.3%)	5 (62.5%)
>0 and < + 1 octave step	> 0 and < + 0.3	Minimal change	9 (50.0%)	3 (37.5%)
0 octave step	0	No change	3 (16.7%)	0 (%)
>−1 and <0 octave step	>−0.3 and < 0	Minimal worsening	0 (%)	0 (%)
≤−1 and >−2 octave steps	≤−0.3 and > −0.6	Mild to moderate worsening	0 (%)	0 (%)
≤−2 octave steps	≤−0.6	Clinically meaningful worsening	0 (%)	0 (%)

Changes were calculated as post-intervention (8 weeks) minus baseline values, with positive values indicating improvement (reduction in stereoacuity threshold) and negative values indicating worsening. Changes were categorized into clinically meaningful, mild-to-moderate, minimal, or no change based on predefined octave and log arc-second thresholds. Data are presented as number of participants (%). Results are shown for the total cohort (*n* = 18) and the subnormal stereoacuity subgroup (*n* = 8).

Participants were stratified into two groups based on baseline stereoacuity: *normal* (<50 arcseconds; *n* = 10) and *subnormal* (≥50 arcseconds; *n* = 8). It was hypothesized that 8 weeks of dichoptic VPL training would result in significant improvement in near stereoacuity.

Secondary outcomes included the change in the number of participants classified as having normal or subnormal near stereoacuity after 8 weeks, based on the Titmus Stereo test. Additionally, changes in distance binocular sensory status were evaluated using the W4D Test, specifically the proportion of participants who transitioned from diplopia or suppression to fusion following the 8-week dichoptic VPL training.

### Statistical analysis

Normality of continuous data was assessed using the Shapiro–Wilk test. To assess potential attrition bias, baseline characteristics were compared between study completers (*n* = 18) and dropouts (*n* = 2) using Mann–Whitney U test and fisher's exact test, respectively.

Outcomes were compared between baseline and 8-week follow-up for both the full cohort (*n* = 18) and a predefined subgroup with subnormal baseline stereoacuity (*n* = 8).

Given the non-normal distribution of stereoacuity (log arcsec), changes over time were analyzed using repeated measures linear regression with generalized estimating equations (GEE) using an identity link function and an exchangeable working correlation structure to account for within-subject correlation. Robust standard errors were used.

Categorical outcomes—such as classification by stereoacuity (normal vs. subnormal) and binocular sensory status (fusion vs. non-fusion)—were analyzed using repeated measures logistic regression with GEE, employing a logit link function, an exchangeable working correlation structure, and robust standard errors.

GEE was used to estimate population-averaged effects while accounting for within-subject correlation across continuous and binary outcomes.

Given the exploratory nature of this study, which evaluated multiple outcomes and subgroup effects, formal correction for multiple comparisons was not applied. Accordingly, findings should be interpreted as hypothesis-generating rather than confirmatory.

All statistical tests were two-tailed with significance set at *P* < 0.05. Analyses were conducted using Stata (StataCorp, College Station, TX) and independently verified by investigators not affiliated with the study sponsor to minimize potential bias.

## Result

### Participants

A total of 20 patients were initially recruited; of these, 18 participants (90.0%) completed the full 8-week study protocol, while two withdrew prior to the baseline assessment due to logistical time constraints (*n* = 1) and a pre-scheduled surgical procedure unrelated to the study (*n* = 1).

Table 2 presents the baseline characteristics of the 18 participants who completed the study. The mean age was 8.1 ± 2.0 years (range, 6–13 years), and the sex distribution was equal (9 females, 9 males). Seventeen participants (94.4%) had the basic type of IXT, while one (5.6%) had the CI type. Fusion was observed in 7 participants (38.9%), diplopia in 4 (22.2%), and suppression in 7 (38.9%). Near stereoacuity was normal (<50 arcsec) in 10 participants (55.6%) and subnormal (≥50 arcsec) in 8 (44.4%). Refractive errors and BCVA for each eye are detailed in Table 2.

**Table 2:** Baseline demographic and clinical characteristics of study participants.

Baseline characteristics	Patients with IXT (*n* = 18)
Demographics	
Age, years	8.1 ± 2.0
Female sex	9 (50)
Type of IXT	
Basic	17 (94.4)
Convergence insufficiency	1 (5.6)
Spherical equivalent refractive errors (Diopters)	
Right Eye	−1.42 ± 1.84
Left Eye	−1.33 ± 1.94
Best-corrected visual acuity (LogMAR)	
Right Eye	0.02 ± 0.07
Left Eye	0.04 ± 0.06
Near stereoacuity (Log arcsec)	1.62 ± 0.24
Normal stereoacuity (< 50 arcsec)	10 (55.6)
Subnormal stereoacuity (≥ 50 arcsec)	8 (44.4)
Distance binocular sensory status	
Fusion	7 (38.9)
Diplopia	4 (22.2)
Suppression	7 (38.9)

Data are presented as no. (%) or mean ± standard deviations. IXT, intermittent exotropia.

To assess potential attrition bias, baseline characteristics were compared between participants who completed the study (*n* = 18) and those who withdrew (*n* = 2). The two participants who withdrew had a mean age of 7.5 ± 3.5 years (*P* = 0.663) and a mean near stereoacuity of 1.75 ± 0.35 log arcsec (*P* = 0.642). Both participants were female (*P* = 0.479) and were diagnosed with the basic type of IXT (*P* > 0.999). Distance binocular sensory status was classified as suppression in both cases (*P* = 0.668). No statistically significant differences were observed between completers and participants who withdrew across any baseline characteristics, suggesting that attrition was unlikely to introduce systematic bias.

### Treatment adherence

Adherence to the prescribed 8-week dichoptic VPL training was high. Among participants who completed the study (*n* = 18), the mean number of completed sessions was 43.8 ± 12.8 sessions, exceeding the minimum requirement of 40 sessions. The mean cumulative treatment time was 411.6 ± 212.7 min, corresponding to 102.9% of the prescribed duration (400 min).

### Changes in near stereoacuity

**Figure 2:**
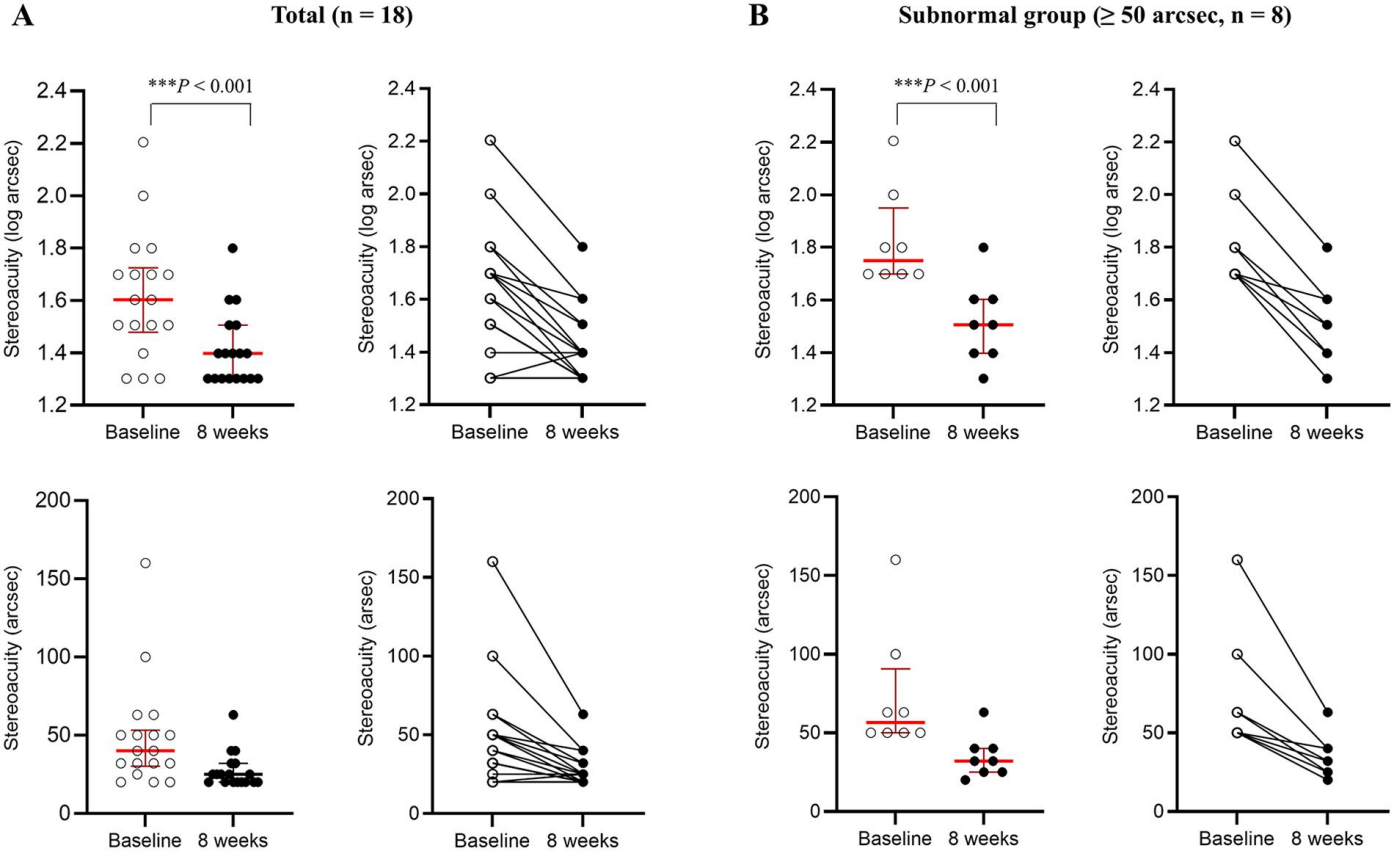
Changes in near stereoacuity after 8 weeks of dichoptic visual perceptual learning. White circles and dark circles represent near stereoacuity measured at baseline and after 8 weeks, respectively (log arcsec in the upper panels; arcsec in the lower panels). Thick dark red horizontal lines indicate medians, while light red lines represent interquartile ranges (25%–75%). Thin connecting lines show within-subject changes over time. **(A)** Among all participants (*n* = 18), near stereoacuity improved significantly after an 8-week dichoptic visual perceptual learning, with a median reduction of 0.20 log arcsec and 13.5 arcsec (*β* = –0.21; ***P < 0.001). **(B)** In the subgroup with subnormal baseline stereoacuity (≥ 50 arcsec, *n* = 8), greater improvement in near stereoacuity was observed after an 8-week dichoptic visual perceptual learning, with a median reduction of 0.35 log arcsec and 30.5 arcsec (*β* = –0.31; ***P < 0.001). *P < 0.05, **P < 0.01, and ***P < 0.001.

Across all participants, near stereoacuity decreased significantly after 8 weeks of dichoptic VPL training, with a mean reduction of 0.21 log arcsec [*β* = –0.21; robust standard error [SE] = 0.04; z = –5.65; 95% confidence interval [CI], −0.28 to −0.13; *P* < 0.001], corresponding to a large within-subject effect size (Cohen's d = 1.33) (Table 3, Figure 2A). Based on octave step classification (Table 1), six participants (33.3%) demonstrated an a mild-to-moderate improvement, corresponding to an increase of ≥1 and <2 octave steps (≥0.3 to <0.6 log arcsec). Nine participants (50.0%) showed minimal improvement (<1 octave step; <0.3 log arcsec) and three participants (16.7%) reported no change (Table 1).

**Table 3:** Changes in near stereoacuity and distance binocular sensory Status after 8 weeks of dichoptic visual perceptual learning.

Clinical characteristics	Baseline	8 weeks	*P* value
Subgroup based on near stereoacuity			0.012
Normal stereoacuity (<50 arcsec)	10 (100)	17 (94.4)	
Subnormal stereoacuity (≥50 arcsec)	8 (0)	1 (5.6)	
Titmus Stereo test (Log arcsec)			
Near stereoacuity score (total, *n* = 18)	1.60 (1.51–1.70)	1.40 (1.30–1.51)	<0.001
Near stereoacuity score (subnormal, *n* = 8)	1.75 (1.70–1.90)	1.51 (1.40–1.60)	<0.001
Worth 4 Dot test			
Total (<50 arcsec, *n* = 18)			0.016
Fusion	7 (38.9)	14 (77.9)	
Diplopia	4 (22.2)	1 (5.6)	
Suppression	7 (38.9)	3 (16.7)	
Subnormal group (≥50 arcsec, *n* = 8)			0.017
Fusion	2 (25.0)	7 (87.5)	
Diplopia	2 (25.0)	1 (12.5)	
Suppression	4 (50.0)	0 (0)	

Continuous outcomes (near stereoacuity scores) were analyzed using repeated-measures linear regression, with an identity link function, whereas categorical outcomes (normal vs. subnormal stereoacuity; fusion vs. non-fusion) using repeated-measures logistic regression, employing a logit link function. All models were implemented via generalized estimating equations with an exchangeable working correlation structure and robust standard errors to account for within-subject correlations. Data are presented as no. (%) or median (25%–75% interquartile range).

Among participants with subnormal stereoacuity at baseline (*n* = 8), the improvement was more pronounced. After 8 weeks, mean stereoacuity decreased by 0.31 log arcsec (*β* = –0.31; robust SE = 0.04; z = –7.67; 95% CI, −0.39 to −0.23; *P* < 0.001), representing a larger within-subject effect size (Cohen's d = 2.71) (Table 3 and Figure 2B). Within this subgroup, five participants (62.5%) demonstrated a mild-to-moderate improvement, corresponding to an increase of ≥1 and <2 octave steps, while three participants (37.5%) showed minimal improvement (<1 octave step) (Table 1).

### Changes in status of near stereoacuity

Based on baseline Titmus Stereo Test results, participants were categorized as having normal stereoacuity (<50 arcsec; *n* = 10, 55.6%) or subnormal stereoacuity (≥50 arcsec; *n* = 8, 44.4%).

At the 8-week follow-up, 17 of 18 participants (94.4%) demonstrated normal stereoacuity, while 1 (5.6%) remained subnormal (Table 3). Logistic GEE analysis showed that the odds of achieving normal stereoacuity were 13.6 times higher at 8 weeks compared with baseline [odds ratio (OR) = 13.6; 95% CI, 1.77–104.3; *β* = 2.61; robust SE = 1.04; z = 2.51; *P* = 0.012], with marginal effects indicating an absolute 38.9% increase in the probability of normal stereoacuity.

Among those with subnormal stereoacuity at baseline, 7 of 8 participants (87.5%) improved to normal, with no instances of deterioration.

### Changes in distance binocular sensory status

**Figure 3:**
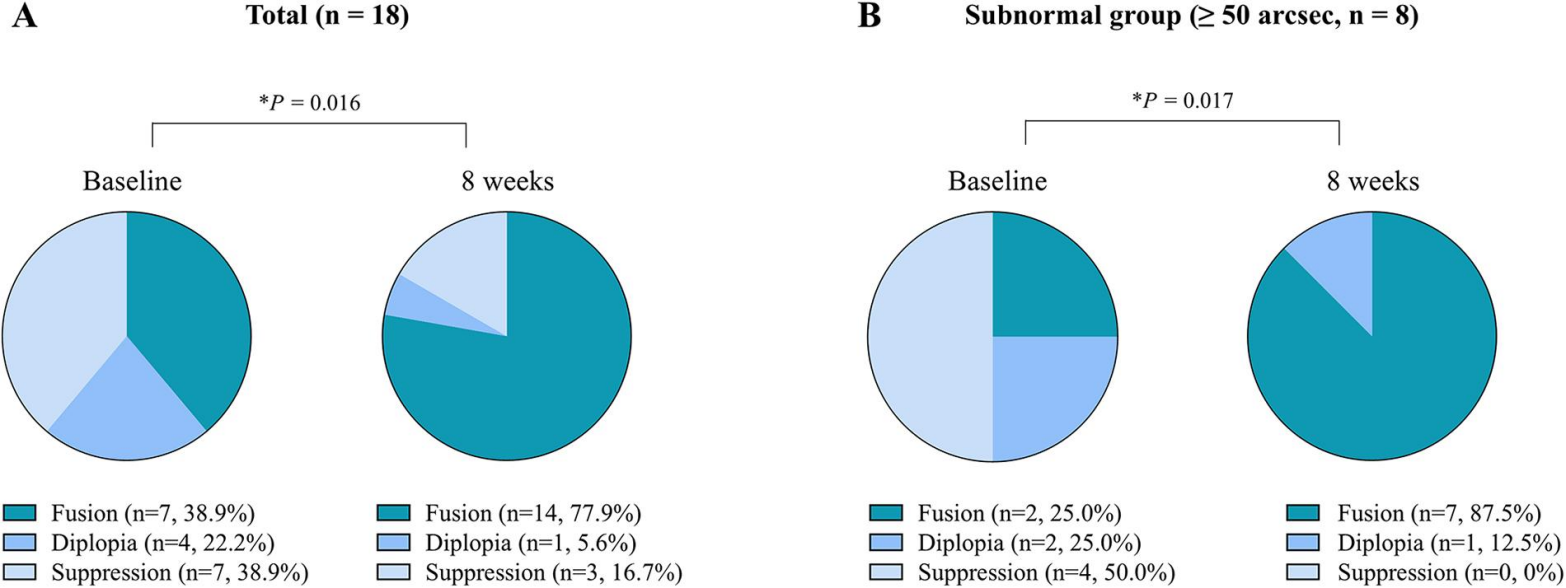
Changes in distance binocular sensory status after 8 weeks of dichoptic visual perceptual learning. Distribution of binocular vision status—fusion, diplopia, and suppression—at baseline and 8 weeks, shown as pie charts with differential colors representing each category. Values indicate the number and percentage of participants at each time point. **(A)** In all participants (*n* = 18), the odds of achieving fusion were 5.5 times higher at 8 weeks compared to baseline (OR = 5.50; *P = 0.016). **(B)** In participants with subnormal baseline stereoacuity (≥ 50 arcsec, *n* = 8), the odds of achieving fusion at 8 weeks were 21 times higher compared to baseline (OR = 21.0; *P = 0.017). *P < 0.05, **P < 0.01, and ***P < 0.001.

GEE analysis revealed a significant improvement in fusion status over the 8-week training period (Table 3). At follow-up, the odds of achieving fusion were 5.50 times higher compared with baseline (OR = 5.50; 95% CI, 1.38–22.0; *β* = 1.70; robust SE = 0.71; z = 2.41; *P* = 0.016; Figure 3A), with marginal effects indicating an absolute 38.9% increase in the probability of fusion. Eight participants (44.4%) transitioned from non-fusion to fusion, including 2 (11.1%) from diplopia and 6 (33.3%) from suppression. One participant (5.6%) showed deterioration, transitioning from fusion to suppression (Table 4).

**Table 4:** Changes between fusion, suppression, and diplopia after 8 weeks of dichoptic visual perceptual learning.

Baseline	8 weeks	Total (*n* = 18)	Subnormal (*n* = 8)
Fusion	Fusion	6 (33.3%)	2 (25.0%)
Diplopia		2 (11.1%)	1 (12.5%)
Suppression		6 (33.3%)	4 (50.0%)
Fusion	Suppression	1 (5.6%)	
Diplopia		1 (5.6%)	
Suppression		1 (5.6%)	
Fusion	Diplopia		
Diplopia		1 (5.6%)	1 (12.5%)
Suppression			

Data are presented as no. (%).

In the subnormal stereoacuity subgroup (*n* = 8), improvement in fusion was more pronounced. At 8 weeks, the odds of achieving fusion were 21.0 times higher than at baseline (OR = 21.0; 95% CI, 1.72–258.0; β = 3.04; robust SE = 1.28; z = 2.38; *P* = 0.017; Figure 3B), with no cases of deterioration. Marginal effects indicated an absolute 62.5% increase in the probability of fusion. Five participants (62.5%) transitioned from non-fusion to fusion—1 (12.5%) from diplopia and 4 (50.0%) from suppression. Two participants (25.0%) showed no change in fusion status, and 1 (12.5%) remained in diplopia (Table 4).

## Discussion

In this study, improvements in near stereoacuity and distance binocular sensory status were observed over an 8-week period in children with IXT undergoing a home-based VR dichoptic VPL program tailored to individual deviation angles in children with IXT. Notably, improvements were more pronounced among participants with subnormal baseline stereoacuity, underscoring the importance of tailoring therapy based on initial sensory function. This personalized, non-invasive digital intervention presented eye-specific stimuli aligned with each individual's ocular deviation, thereby enhancing both stereopsis and sensory fusion.

After eight weeks of training, 44.4% of all participants and 62.5% of those with subnormal stereoacuity transitioned from diplopia or suppression to fusion. These findings highlight that homogeneous retinal correspondence and stable binocular sensory status are essential prerequisites for the development of stereopsis. In contrast, asymmetric retinal correspondence—often associated with diplopia or suppression—disrupts stereopsis (21, 22). In IXT, delayed development of motor fusion and impaired sensory fusion due to abnormal retinal correspondence are established contributors to poor binocular function (23–25). Achieving fusion in IXT requires both ocular alignment and increased accommodative effort to maintain binocular single vision, reflecting the elevated demand on fusional mechanisms in these individuals (26).

The dichoptic VPL program used in this study delivered eye-specific stimuli via a VR headset calibrated to each participant's deviation angle. This approach may promote binocular simultaneity perception and enhance sensory fusion, thereby improving stereopsis (27, 28). The home-based training regimen leveraged visual system plasticity through repeated depth discrimination tasks designed to facilitate VPL (12, 13, 15). Although the precise neural mechanisms remain to be fully elucidated, previous studies suggest that VPL induces changes in cortical connectivity, as observed in cases of cortical blindness (29, 30). By generating binocular disparity cues through eye-specific stimulation, this personalized digital therapy engages neuroplastic processes that support fusion and depth discrimination—specifically, the perception of convexity in depth, a key marker of binocular function (31–34). Prior research has demonstrated that VR-based stereoscopic vision training can enhance normal retinal correspondence, fusion ability, and stereoacuity (35–38). Similarly, VPL-based visual training has restored simultaneity perception, fusion, and stereopsis in children with IXT following surgery (39). These findings are consistent with our results and support the efficacy of personalized dichoptic VPL programs in improving stereopsis by targeting binocular integration mechanisms and harnessing visual system plasticity.

This immersive, home-based digital therapy shows strong potential to enhance adherence, increase treatment efficacy, and broaden clinical applicability in managing childhood IXT (16). While traditional patching therapy remains accessible and personalized, it is limited by short-term efficacy, low engagement, and its repetitive nature (40). In contrast, VR-based interventions offer dynamic visual feedback and immersive environments with precise head-tracking, minimal side effects, and fewer spatial or temporal constraints—features that may particularly benefit pediatric adherence and engagement (16). Improvements in binocular alignment through dichoptic oculomotor training further highlight VR-based therapy as a promising non-surgical alternative for managing ocular alignment disorders (41).

The absence of a no-training or sham-training control group and the lack of distance stereopsis measurements should be considered when interpreting these results. This study primarily assessed the therapeutic efficacy of a VR-based dichoptic VPL program for childhood IXT, a condition with few effective long-term, non-invasive treatment options. Given the small sample size and the absence of a control group, the current findings should be interpreted with caution. Moderate improvements were observed in 33.3% of all participants and 62.5% of those with baseline deficits, with no evidence of symptom deterioration following training. Nevertheless, improvements in near stereopsis did not reach the ≥2-octave threshold for clinical significance. This limited improvement may reflect a ceiling effect due to relatively preserved baseline stereoacuity in a substantial proportion of participants, along with the short 8-week training period. Improvements in stereopsis and binocular sensory function following VR-based dichoptic VPL may partially reflect learning effects, regression to the mean, test repeatability, or natural fluctuations inherent to IXT. Given the multiple outcomes and subgroup comparisons examined, this study was exploratory in nature, and the results should be interpreted cautiously pending confirmation in larger, controlled trials.

Moreover, the single-arm pre–post design precluded masked assessment, which may have introduced assessment bias, although all evaluations were conducted using standardized testing procedures to minimize this risk. Future large randomized controlled trials incorporating a no-intervention or sham-treated control group, along with masked assessment, will provide more robust evidence of efficacy and help elucidate the underlying mechanisms contributing to stereopsis enhancement. Additionally, the present study evaluated only near stereoacuity, despite distance stereopsis being a key functional domain in IXT, where distance control is more impaired. Moreover, near stereopsis was assessed using the Titmus Stereo Test, which has inherent limitations, including relatively coarse disparity steps and the presence of monocular cues in certain plates. Future research should evaluate the impact of dichoptic VPL training on distance vision and stereopsis using highly validated measures to provide a more comprehensive assessment of treatment benefit.

Longitudinal studies involving larger, more diverse populations, including adults with IXT, are also warranted to validate these findings and enhance their generalizability. Such studies could further investigate the durability of treatment effects, optimal training duration, and the potential benefits of tailoring therapy intensity based on initial sensory status. Our findings suggest that an 8-week, home-based VR dichoptic VPL program—customized to each child's deviation angle—can improve near stereoacuity and distance binocular sensory function in children with IXT. Notably, participants with subnormal baseline stereoacuity demonstrated greater improvements, suggesting that individualized treatment parameters may further optimize outcomes in this subgroup.

As a non-invasive, engaging, and scalable intervention, VR-based dichoptic VPL therapy shows considerable promise for binocular vision rehabilitation. By targeting neuroplasticity and leveraging an immersive digital format that may enhance adherence, this approach has the potential to improve treatment outcomes and broaden clinical application in the management of IXT.

## Data Availability

The datasets presented in this article are not readily available because the datasets generated and analyzed in this study contain sensitive human participant data and are therefore not publicly available due to ethical and privacy restrictions imposed by the Institutional Review Board. Access to de-identified data may be considered upon reasonable request and subject to IRB approval. Requests to access the datasets should be directed to Dong-Wha Kang, dwkang@amc.seoul.kr.

## Author disclaimer

Nunaps Inc. (D-W.K) sponsored the research and contributed to the study design, as well as development and validation of the digital therapeutic intervention. The sponsor also reviewed, and approved the manuscript; and made the decision to submit the manuscript for publication. Data collection and interpretation were performed by ophthalmologists (B.J.L.) and researchers (H.R., J.W.L.) at Asan Medical Center in accordance with institutional protocols and independently of the sponsor (Nunaps Inc.). Statistical analyses and manuscript preparation were conducted independently by non-sponsor-affiliated investigators (E.N. and H.R.) at Asan Medical Center.
